# Lymph node ratio is inferior to pN-stage in predicting outcome in colon cancer patients with high numbers of analyzed lymph nodes

**DOI:** 10.1186/s12893-018-0417-0

**Published:** 2018-10-03

**Authors:** Manuel O Jakob, Ulrich Guller, Alex Ochsner, Daniel Oertli, Markus Zuber, Carsten T Viehl

**Affiliations:** 1 Department of Surgery, Spitalzentrum Biel-Bienne AG, Vogelsang 84, CH-2501 Biel, Switzerland; 2 Department of Oncology, Kantonsspital St. Gallen, St. Gallen, Switzerland; 3 Department of Surgery, Spital Limmattal, Schlieren, Switzerland; 4 Department of Surgery, University of Basel, Basel, Switzerland; 5 Department of Surgery, Kantonsspital Olten, Olten, Switzerland

**Keywords:** Lymph node ratio, pN-stage, Outcome, Colon cancer

## Abstract

**Background:**

The lymph node ratio (LNR), i.e. the number of positive lymph nodes (LN) divided by the total number of analyzed LN, has been described as a strong outcome predictor in node-positive colon cancer patients. However, most published analyses are constrained by relatively low numbers of analyzed LN. Therefore, the objective of the present study was to evaluate the prognostic impact of LNR in colon cancer patients with high numbers of analyzed LN.

**Methods:**

One hundred sixty-six colon cancer patients underwent open colon resection. All node-positive patients were analyzed for this study. The number of analyzed LN, of positive LN, the disease-free (DFS) and overall survival (OS) time were prospectively recorded. Patients were dichotomously allocated to a high or a low LNR-group, respectively, with the median LNR (0.125) as a cut-off value. Median follow-up was 34.3 months.

**Results:**

Fifty-eight patients (34.9%) were node-positive. The median number of analyzed LN was 23 (range 8–54). DFS and OS were significantly shorter in pN2 vs pN1 patients (*p* < 0.001, and *p* = 0.001, respectively), and in LNR high vs low patients (*p* = 0.032, and *p* = 0.034, respectively). pN2 (vs pN1) disease showed hazard ratios (HR) of 6.2 (*p* < 0.001), and 6.8 (*p* < 0.005; for DFS and OS, respectively), while LNR high (vs low) showed HR of 3.0 (*p* =0.041), and 4.5 (*p* = 0.054).

**Conclusions:**

LNR is a reasonable outcome predictor in node-positive colon cancer patients. However, LNR is inferior to pN-stage in predicting survival in patients with high number of harvested lymph nodes.

**Electronic supplementary material:**

The online version of this article (10.1186/s12893-018-0417-0) contains supplementary material, which is available to authorized users.

## Background

Colorectal cancer is one of the most common cancers worldwide. Surgery remains the mainstay of treatment in most colon cancer patients. Outcome prediction is based on the tumor-node-metastasis (TNM) system reflected by the American Joint Committee on Cancer and the International Union against Cancer (AJCC/UICC) [1, 2]. Metastases to regional lymph nodes (LN) is a strong outcome predictor following surgical resection [3]. Moreover, LN status is the most important determinant whether adjuvant chemotherapy should be given or not. Patients with positive LN involvement are classified as stage III if no distant metastasis can be detected. Further subclassification into pN1-, and pN2-category, respectively, is based on the number of LN involved. The AJCC guidelines recommend at least 12 LN harvested and analyzed in the surgical specimen as a quality indicator for adequate staging [1, 2]. Most importantly, low LN harvest may lead to inadequate nodal staging which in turn may result in undertreatment of patients who would potentially benefit from adjuvant chemotherapy. Additionally, survival improves with higher numbers of analyzed LN in stage I, II and III patients [4]. Recent studies have recommended the lymph node ratio (LNR), i.e. the number of positive LN divided by the number of analyzed LN, as a stronger indicator than pN-category in predicting survival in colon cancer patients [5–7]. However, most of the published analyses have been conducted on series with relatively low total numbers of analyzed LN and/or on mixed series of colon and rectal cancer patients. Therefore, the objective of the present study was to compare the prognostic impact of LNR and pN-classification in a series of colon cancer patients with a median number of analyzed LN almost double than the demanded minimum.

## Methods

### Study design

The current multicenter study is based on the „Swiss prospective, multicenter study Sentinel Lymph Node Procedure in Colon Cancer“ (NCT00826579) [8] and was conducted in three academic or university-affiliated hospitals in Switzerland (University Hospital Basel, Kantonsspital Olten, Spitalzentrum Biel). The data was prospectively recorded in a database (Microsoft Office Excel 2007) from 2000 through 2005. One hundred and sixty-six patients with biopsy proven colon cancer of AJCC stages I-III underwent open, oncologic colon resections according to the tumor location and en-bloc oncologic LN dissection. At this point in time laparoscopic resections were not yet performed at our institutions for cancer cases. Patients underwent surveillance according to national guidelines and were additionally followed by chart review, questionnaires, and phone interviews.

Patients presenting with stage I and II colon cancers were excluded for this study and the remaining stage III colon cancer patients were analyzed further (*n* = 58). Patient’s characteristics are given in Table 1. Further exclusion criteria were distant metastases, rectal cancers, prior abdominal cancer surgery, history of other malignancies, allergy to isosulfan blue, pregnancy, and breast-feeding. Distant metastases were excluded by preoperatively performing chest X-ray or CT-scan, abdominal CT-scan and/or an abdominal ultrasound. The formalin-fixed specimen was reviewed twice by a dedicated pathologist using macroscopic and microscopic examination.

**Table 1:** Clinical data of all stage III patients

	Total	pN1	pN2	*p* value	LNR low	LNR high	*p* value
	*n* = 58^a^ (100%)	*n* = 38^a^ (65.5%)	*n* = 20^a^ (34.5%)		*n* = 26^a^ (44.8%)	*n* = 32^a^ (55.2%)	
Age^b^ (years)	71 (65–78)	71 (63–77)	73 (65–80)	0.330	66 (56–77)	72 (67–79)	0.050
Gender							
- Male	36 (62.1)	22 (57.9)	14 (70.0)	0.408	15 (57.7)	21 (65.6)	0.594
- Female	22 (37.9)	16 (42.1)	6 (30.0)		11 (42.3)	11 (34.4)	
Localization							
- Right colon	24 (41.4)	16 (42.1)	8 (40.0)	0.784	11 (42.3)	13 (40.6)	0.624
- Transverse colon	1 (1.7)	1 (2.6)	–		1 (3.8)	–	
- Left colon	15 (25.9)	9 (23.7)	6 (30.0)		6 (23.1)	9 (28.1)	
- Sigmoid colon	18 (31.0)	12 (31.6)	6 (30.0)		8 (30.8)	10 (31.3)	
T category							
- pT1	3 (5.2)	3 (7.9)	–	0.050	1 (3.9)	2 (6.2)	0.539
- pT2	4 (6.9)	4 (10.5)	–		3 (11.5)	1 (3.1)	
- pT3	42 (72.4)	28 (73.7)	14 (70.0)		19 (73.1)	23 (71.9)	
- pT4	9 (15.5)	3 (7.9)	6 (30.0)		3 (11.5)	6 (18.8)	
Tumor grading							
- G1	–	–	–	0.055	–	–	0.543
- G2	44 (75.9)	32 (84.2)	12 (60.0)		21 (80.8)	23 (71.9)	
- G3	14 (24.1)	6 (15.8)	8 (40.0)		5 (19.2)	9 (28.1)	
AJCC stage							
- IIIa	7 (12.1)	7 (18.4)	–	< 0.001	4 (15.4)	3 (9.4)	< 0.001
- IIIb	32 (55.2)	31 (81.6)	1 (5.0)		22 (84.6)	10 (21.2)	
- IIIc	19 (32.7)	–	19 (95.0)		–	19 (59.4)	
Total number of LN analyzed^b^	23 (14–32)	21 (14–29)	25 (18–37)	0.149	25 (19–31)	21 (13–32)	0.159
Patients with adjuvant chemotherapy	43 (74.1)	29 (76.3)	14 (70.0)	0.753	19 (73.1)	24 (75.0)	1.000
Center							
- Olten	33 (56.9)	18 (47.4)	15 (75.0)	0.130	15 (57.7)	18 (56.3)	0.416
- Biel	15 (25.9)	12 (31.6)	3 (15.0)		5 (19.2)	10 (31.2)	
- Basel	10 (14.2)	8 (21.0)	2 (10.0)		6 (23.1)	4 (12.5)	

^a^With percentages in parentheses; ^b^values are median (interquartile range)

The following information was collected: patients` characteristics, operation performed, number of resected LN, number of metastatic LN, TNM-stage, LNR, grading (well, moderately, poorly differentiated tumors), adjuvant chemotherapy, cancer recurrence, postoperative complications, death. Disease-free survival (DFS) was defined as the time from the date of surgery to the diagnosis of cancer recurrence or death for any cause, whichever occurred first. Overall-survival (OS) was specified as the time from the date of surgery to death for any cause.

For the analysis of the LNR we defined two groups: Patients were dichotomously allocated to a high or a low LNR-group, respectively, with the median LNR (0.125) as a cut-off value. We choose the median LNR as cut-off to have a balanced patients’ distribution and enough power to compare the two LNR-groups with pN-stage: LNR low group included all patients with LNR < 0.125; LNR high group all patients LNR ≥ 0.125.

### Ethical approval and consent to participate

The study has been approved by the regional Ethic committees of Basel (#201/00), Bern (#30/2001), and Olten (#EKO-0026). A written consent was signed by every participant.

### Statistical analysis

Statistical analyses were performed using NCSS Version 9.0.9 software (2013, Kaysville, Utah, USA). Patients’ demographic data were examined and reported using descriptive analyses. The Fishers exact test for categorical variables as well as Mann-Whitney U test for continuous variables were used for exploratory comparisons of patients’ groups. Univariable survival analysis was performed using the Kaplan-Meier method to compare the survival in patients’ subgroups and the log-rank test for statistical comparison. Cox regression was used to detect whether LNR (low vs. high) and pN-stage are prognostic factor regarding to OS and DFS. Results are reported as hazard ratio (HR) with 95% confidence interval (95% CI) and a *P*-value. Continuous explanatory variables (LNR) were dichotomized based on the median. A two-sided *p*-value of < 0.05 was chosen to indicate statistical significance.

## Results

Fifty-eight patients were node positive and classified into UICC/AJCC stage III. Median follow-up was 34.3 months (95% CI 24.4–41.1). No patient was lost to follow-up. The median number of harvested lymph nodes in this group was 23 (mean 23.8, range 8–54). The median number of positive lymph nodes was three (range 1–19). Only three patients (5.2%) had fewer than twelve LN analyzed: two right-sided hemicolectomies (10, and 11 LN, respectively) and one rectosigmoid resection (8 LN). Median LN count in the surgical specimens was: right hemicolectomy (*n* = 18) 22, extended right hemicolectomy (*n* = 6) 37, resection of the transverse colon (*n* = 1) 27, left hemicolectomy (*n* = 12) 18, extended left hemicolectomy (*n* = 3) 32, sigmoid resection (*n* = 5) 25, rectosigmoid resection (*n* = 13) 22. Early complications (<30d) included patients with intraoperative (*n* = 4, 6.9%) and postoperative complications (*n* = 10, 17.2%) during the hospital stay. Two patients had an anastomotic leakage (3.5%). Late complications included two patients (3.4%) with ileus and one patient (1.7%) with symptomatic incisional hernia. All node positive patients were considered for adjuvant chemotherapy at the respective multidisciplinary meetings. Finally, 43 patients received adjuvant chemotherapy (74.1%); the remaining patients were not considered adequately fit for or refused the chemotherapy. Patients receiving adjuvant chemotherapy had a better overall survival compared to patients without chemotherapy (Additional file 1: Figure S1a and b). Thirty-two (55.2%) patients had cancer recurrence during follow up. Median DFS and OS were 24.2 (95% CI 16.1–35.4), and 28.8 month (95% CI 22.1–38.1), respectively.

**Fig. 1:**
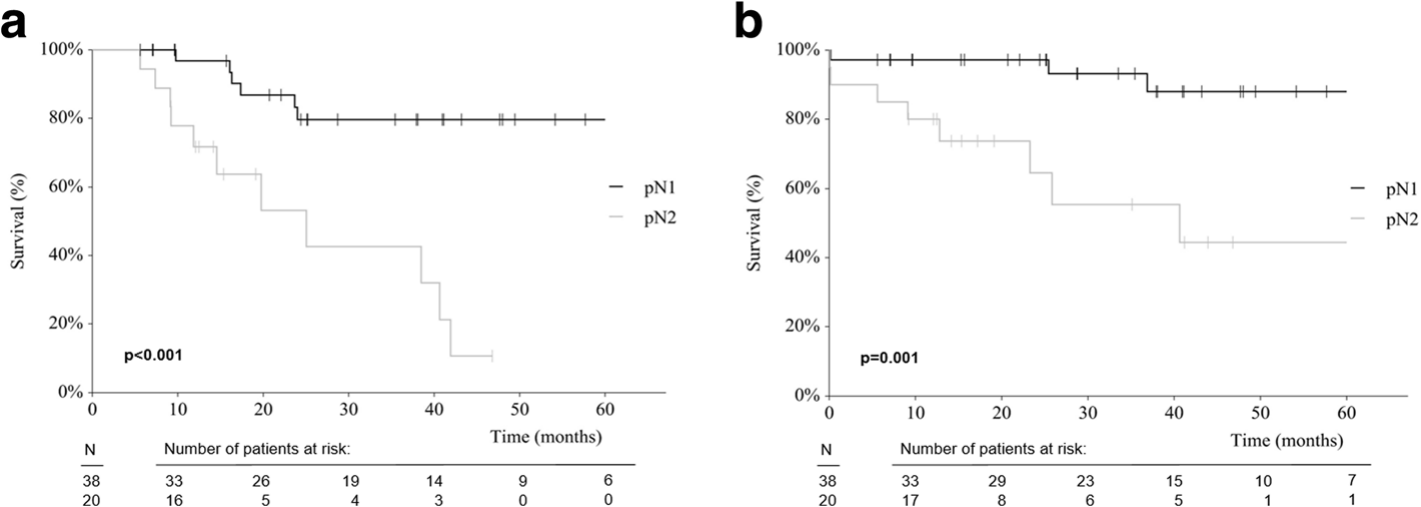
**a** DFS according to pN-category (pN1 vs pN2). **b** OS according to pN- category (pN1 vs pN2)

Of the 58 patients, 38 (65.5%) had pN1 disease and 20 (34.5%) had pN2 disease. Cancer recurrence was significantly more frequent in pN2 than pN1 patients (70.0 vs 39.5%, *p* = 0.002, Table 2). DFS and OS was significantly shorter in pN2 patients compared to pN1 patients (*p* < 0.001, and *p* = 0.001, respectively, Table 2, Figs. 1a-b).

**Table 2:** Cancer recurrence and survival according to pN-category and LNR-groups

	Recurrence free patients at 24 months^a^	Median DFS in months^b^	Median OS in months^b^
pN-category			
1 (*n* = 38)	23 (60.5)	26.9 (17.3–40.9)	34.5 (24.4–41.1)
2 (*n* = 20)	6 (30.0)	13.3 (9.2–19.8)	16.3 (12.1–35.1)
LNR-group			
Low (*n* = 26)	17 (65.4)	26.9 (16.3–43.2)	32.1 (24.4–48.0)
High (*n* = 32)	12 (37.5)	15.0 (9.2–25.0)	19.9 (12.1–38.1)

*DFS* Disease-free survival, *OS* Overall survival, *LNR* Lymph node ratio

^a^With percentages in parentheses, ^b^values are median (95% confidence interval)

**Fig. 2:**
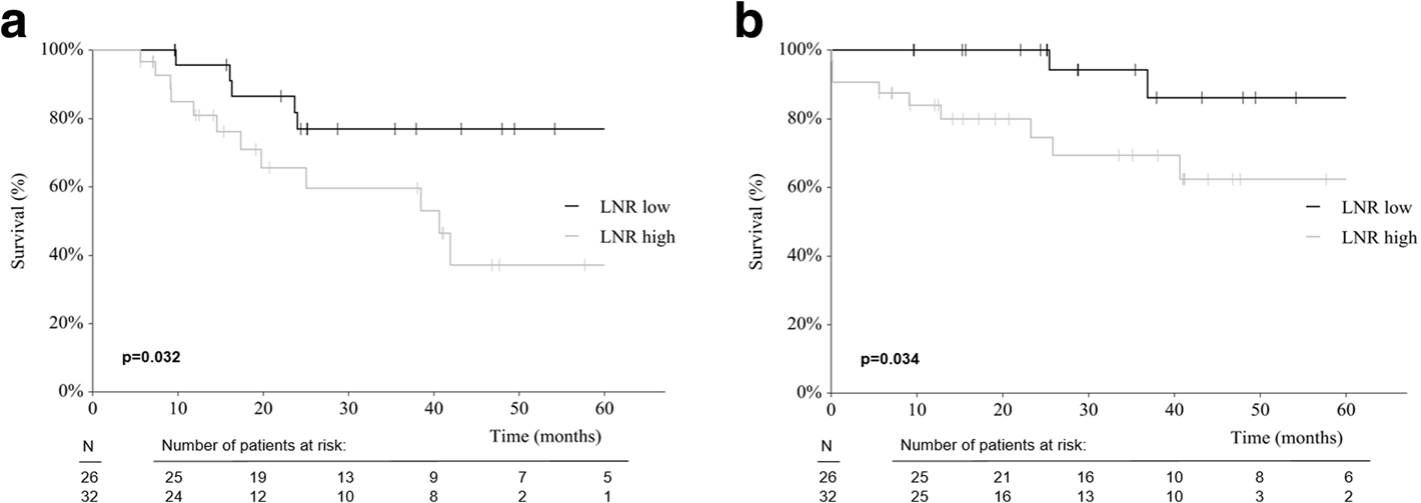
**a** DFS according to LNR (LNR low vs LNR high). **b** OS according to LNR (LNR low vs high)

Of the 58 patients, 26 (45.0%) had a low LNR, and 32 (55.0%) had a high LNR. The LNR high group showed only a trend towards a more frequent cancer recurrence compared to the LNR low group (62.5 vs 34.6%, *p* = 0.128, Table 2). DFS and OS were significantly shorter in the LNR high group compared to the LNR low group. (*p* = 0.032, and *p* = 0.034, respectively, Table 2, Figs. 2a and b).

Cross-table comparison of pN1 vs. pN2 and LNR low vs. LNR high shows the shortest survival time for the combination of pN2 disease and LNR high, and the longest survival for the combination of pN1 disease and LNR high. The combination of pN2 disease and LNR low did not occur in our patient population (Table 3).

**Table 3:** Survival rates according to LNR-groups

	LNR low (*n* = 26)	LNR high (*n* = 32)
pN1^a^	26 (68.4%)	12 (31.6%)
DFS in months^b^	26.9 (16.3–43.2)	29.4 (7.0–47.7)
OS in months^b^	32.1 (24.4–48.0)	35.8 (7.0–47.7)
pN2^a^	–	20 (100%)
DFS in months^b^	–	13.3 (9.2–19.8)
OS in months^b^	–	16.3 (12.1–35.1)

LNR, lymph node ratio; DFS, disease-free survival; OS, overall survival

^a^With percentages in parentheses; ^b^values are median (95% confidence interval)

The comparison of pN1 vs pN2 produced a clearer curve separation in Kaplan-Meier survival curve analysis than the comparison LNR low vs high (Figs. 1 and 2). Similarly, univariate Cox-Regression analysis revealed a higher prognostic power of pN category as compared to LNR in this setting of a high number of analyzed LN (Table 4): pN2 (vs pN1) disease showed hazard ratios (HR) of 6.2 (*p* < 0.001), and 6.8 (*p* < 0.005; for DFS and OS, respectively), while LNR high (vs low) showed HR of 3.0 (*p* = 0.041), and 4.5 (*p* = 0.054). As shown, the LNR analysis did not significantly predict OS. Even when applying our data on published LNR cut-off values (0.17, 0.41, and 0.69), no clear discrimination of survival curves in terms of DFS and OS was found (Additional file 2: Figure S2a and b) [6, 7].

**Table 4:** Univariate Cox Regression Analysis for DFS/OS

	DFS		OS	
	HR^a^	*p* value	HR	*p* value
pN-stage (2 vs. 1)	6.2 (2.3–17.1)	< 0.001	6.8 (1.8–26)	0.005
LNR (high vs. low)	3.0 (1.0–8.5)	0.041	4.5 (1. – 21.1)	0.054

*DFS* Disease-free survival, *OS* Overall survival

^a^HR, hazard ratio (95% confidence interval)

## Discussion

In this prospectively recorded, multi-center study we provide compelling evidence that LNR is inferior to pN-category in predicting recurrence and survival for stage III colon cancer patients with high number of analyzed lymph nodes. The strength of this study is an excellent lymph node staging, as well as no loss to follow-up.

The number of positive LN in the resected surgical specimen (reflected by the pN category) has been stated to be a strong predictor of prognosis in stage III colon cancer [9]. However, recent studies suggested that the LNR might be a more reliable indicator for outcome prediction [5–7]. Since the TNM system disregards the total number of analyzed LN and focuses only on the number of positive LN, this ratio-based classification has been implemented in several studies and new guidelines have been proposed accordingly [10, 11]. However, this does not solve the problem of possible understaging when only an inadequate number of LN is harvested and analyzed. Therefore, if a radical oncologic resection is conducted as well as if an accurate histologic exam is performed, the LNR might be less important than expected. If the number of positive LN increases, the value of LNR will increase, as well. Conversely, the higher the total amount of LN harvested, the lower the LNR will be, even if the number of positive nodes is high. Therefore, high numbers of resected LN might bias the LNR or published cut-off values for LNR might be inaccurate.

Several cut-off values for the LNR have been used in previous studies. De Ridder et al. analyzed 26,181 colorectal cancer patients and chose a cut-off value of 0.4 using Nagelkerke’s r2 index for LNR low vs. high [12]. However, their study is based on a insufficient median LN-number of ten and their prognostic separation using LNR was only slightly better (31%) compared with the UICC pN stage (26%). In our study, we used the median LNR as the cut-off value. The high number of negative LN (because of the high total number of LN analyzed) did lower the median LNR and a cut-off value of ≥0.4 as suggested by De Ridder et al. is therefore reached only by three patients in our study.

A radical surgical resection with high numbers of analyzed LN has been shown to be a most important prognostic factor in colon cancer [13]. Several authors stated that oncologic LN dissection has an important role as quality indicator and as a surrogate marker of a good operation performed [6, 14, 15]. According to Le Voyer et al. the number of LN removed and analyzed is an important parameter that positively affects survival in node-positive and node-negative patients [16]. The higher the amount of resected LN in the surgical specimen the more accurate the examination by the pathologist. Thus, a high amount of resected LN decreases the risk of understaging due to missed involved LN and might lead to stage migration.

Wang et al. found only a median of 11 analyzed LN in 24,477 patients in the SEER registry; they showed that understaging is a serious problem especially in stage IIIb and IIIc patients [10]. Berger et al. stated in the first report on LNR that there are distinct LNR-subgroups within the pN1 and pN2 stages [17]. They found a worse OS for pN1 patients with a higher LNR than for pN2 patients with a lower LNR. In our data, no such effect was detected, and *all* the pN2 patients were found in the LNR high group. More importantly, pN2 patients showed a shorter DFS and OS compared to LNR high group patients. Finally, LNR analysis could not significantly predict cancer recurrence nor was the LNR a significant prognostic parameter for OS in Cox regression analysis. These observations taken together speak for the higher prognostic power of the pN category in the setting of colon cancer patients with high numbers of analyzed LN.

Two studies with high surgical quality and a relatively high amount of LN resected (median 16, and 16.8, respectively) stated that the LNR is a stronger indicator in outcome prediction and a more accurate marker for stratifying prognosis than the AJCC/UICC classification [6, 18]. Even though a high surgical standard is clearly given in these studies, there still is a remarkable difference in LN-count compared to our data. A recent meta-analysis supporting LNR being superior compared to pN-category in outcome prediction mostly included retrospective studies with a low median number of LN resected [19]. Conversely, our findings are supported by Mohan et al. and Schiffmann et al., who did not find a difference in outcome predicting comparing LNR and pN-category, even with a marked lower resected number of lymph nodes compared to our data [20, 21].

The AJCC recommends the examination of at least 12 lymph nodes as an indicator of adequate lymphadenectomy [1]. However, many surgeons and pathologists still do not live up to this expectation, and many published studies have been carried out with much less lymph nodes harvested in the surgical specimen [22, 23]. In our series, an extremely high median number of 23 harvested LN was observed, and 95% of patients were adequately operated with 12 or more LN resected. Additionally, we had no loss to follow-up, which further contributes to the strength of this study.

We would also like to mention the possible limitations of our study: Due to rather small number of node positive patients (even when applying the sentinel procedure in each patient) the generalization of our data has to be used with caution. Additionally, the median follow up was three years; nonetheless tumor recurrence typically occurs within the first two to three years postoperatively.

## Conclusions

In conclusion, our data shows that LNR is a good outcome predictor in node-positive colon cancer patients. However, we were able to show that LNR is inferior to pN-category in predicting recurrence and survival in patients with high number of analyzed lymph nodes. Therefore the prognostic impact of pN category seems to be more important in patients with a high number of analyzed LN and the role of LNR has to be questioned in this setting.

## Additional files

Additional file 1: **Figure S1. a** DFS according to adjuvant chemotherapy. **b** OS according to adjuvant chemotherapy. (PPTX 2560 kb)

Additional file 2: **Figure S2. a** DFS according to published cut-off values. **b** OS according to published cut-off values. (PPTX 2649 kb)
