# Supplementary figures and images for: Lymph node ratio is inferior to pN-stage in predicting outcome in colon cancer patients with high numbers of analyzed lymph nodes
Source: BMC Surg. 2018 Oct 3;18:81. doi: 10.1186/s12893-018-0417-0 (PMC6171184; doi:10.1186/s12893-018-0417-0)

## Slide 1
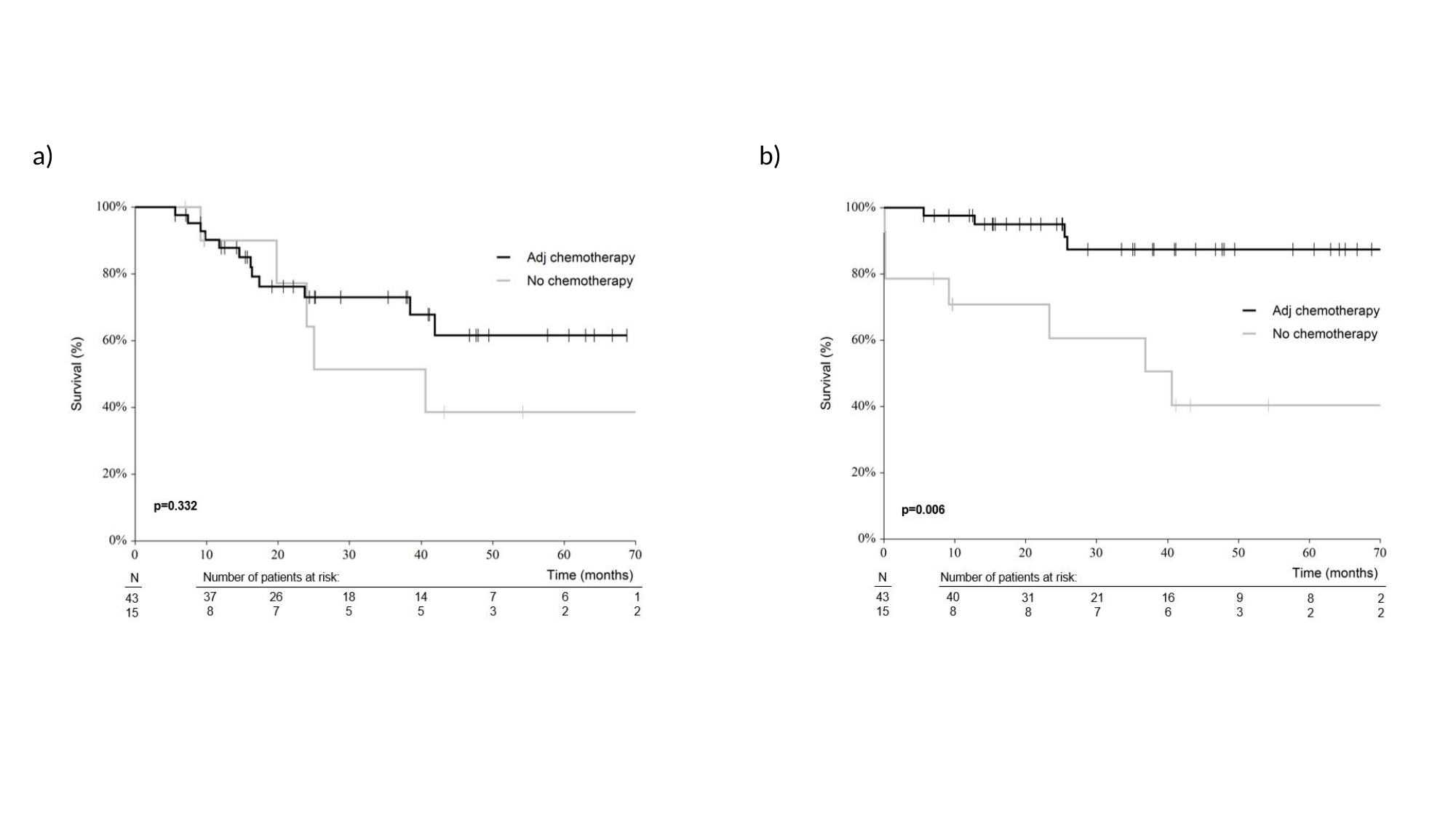

a)
b)

Supplement: Supplementary file 1 — Figure S1. a DFS according to adjuvant chemotherapy. b OS according to adjuvant chemotherapy. (PPTX 2560 kb) [file 12893_2018_417_MOESM1_ESM.pptx]

## Slide 1
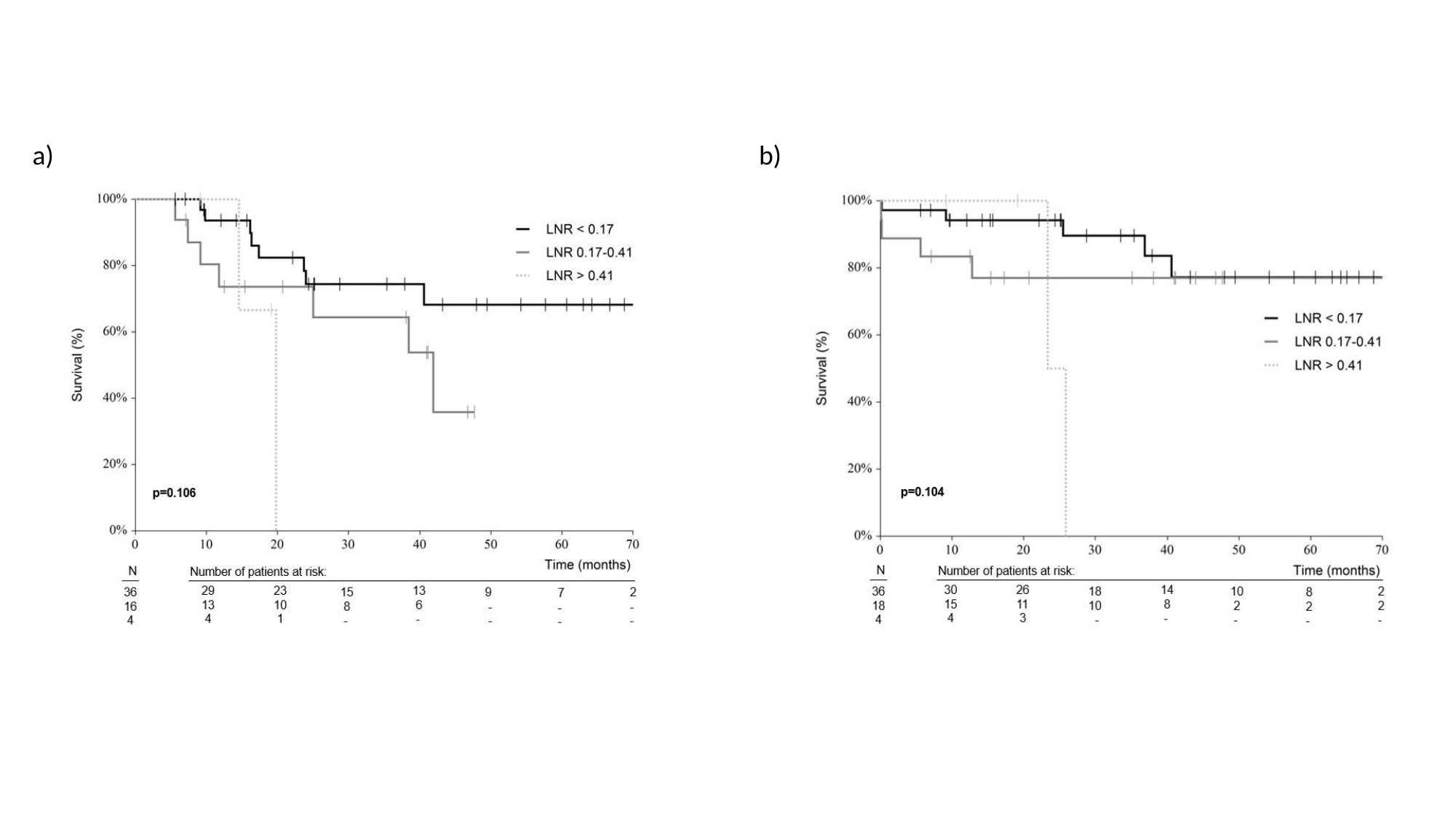

a)
b)

Supplement: Supplementary file 2 — Figure S2. a DFS according to published cut-off values. b OS according to published cut-off values. (PPTX 2649 kb) [file 12893_2018_417_MOESM2_ESM.pptx]
